# Autistic Traits as Predictors of Increased Obsessive–Compulsive Disorder Severity: The Role of Inflexibility and Communication Impairment

**DOI:** 10.3390/brainsci14010064

**Published:** 2024-01-09

**Authors:** Liliana Dell’Osso, Benedetta Nardi, Chiara Bonelli, Giulia Amatori, Maria Alessandra Pereyra, Enrico Massimetti, Ivan Mirko Cremone, Stefano Pini, Barbara Carpita

**Affiliations:** 1 Department of Clinical and Experimental Medicine, University of Pisa, 56126 Pisa, Italy; liliana.dellosso@gmail.com (L.D.); chiarabonelli.95@hotmail.it (C.B.); giulia.amatori@libero.it (G.A.); alessandra.pereyra19@gmail.com (M.A.P.); ivan.cremone@gmail.com (I.M.C.); stefano.pini@unipi.it (S.P.); barbara.carpita@unipi.it (B.C.); 2 UFSMA Val di Cornia, Azienda USL Toscana Nord Ovest, 54100 Massa, Italy; e.massimetti@yahoo.it

**Keywords:** obsessive–compulsive spectrum, autism spectrum disorder, autistic traits, subthreshold autism spectrum, cognitive inflexibility

## Abstract

Due to similar manifestations, some authors have proposed a potential correlation between autism spectrum disorder (ASD) and obsessive–compulsive disorder (OCD). This link has long been recognized and debated, with some authors arguing that these disorders frequently occur comorbid but distinct while others believe they are part of the same spectrum. The aim of our study was to explore the prevalence and correlates of autistic traits in 55 OCD patients and 55 matched controls and to assess possible autistic dimensions predictive of higher OCD symptoms. All participants were assessed with the Obsessive–Compulsive Spectrum-Short Version (OBS-SV) and the Adult Autism Subthreshold Spectrum (AdAS Spectrum). The OCD group scored significantly higher in both questionnaires. Total OBS-SV scores and domains were significantly correlated with all AdAS Spectrum domains and total score. The AdAS Spectrum total, *Verbal Communication* and *Inflexibility and adherence to routine* domain scores were significant positive predictors of higher OBS-SV scores. Lastly, when two clusters of subjects (*high* and *low autism*) were determined, *Inflexibility and adherence to routine domain* presented the greatest influence in forming the clusters. Our findings support the association between OCD and autistic traits in the adult population, supporting the hypothesis of a neurodevelopmental basis for these psychiatric conditions.

## 1. Introduction

Obsessive–compulsive disorder (OCD) is a highly heterogenous, common psychiatric condition with a prevalence rate around 2% and can cause major functional restrictions in affected persons on a daily basis [1,2,3]. According to the World Health Organization (WHO), OCD is among the 10 most disabling medical conditions in the world, and it is also the fourth most common mental disorder [4,5]. The Diagnostic and Statistical Manual of Mental Disorders, Fifth Edition, Text Revision (DSM-5-TR), defines frequent obsessional thoughts and/or compulsive behaviors as the principal hallmarks of OCD [6]. Obsessions are persistent and stereotyped thoughts, pictures or urges, experienced as distressing, acknowledged as belonging to the individual and frequently rejected [7]. These thoughts and images frequently convey thoughts of possible danger to other people or to the individual, like the idea that touching a surface contaminated by germs might result in a deadly illness [6]. Compulsions are repetitive behaviors or mental acts that the person feels compelled to perform in response to an obsession, following rigid rules, whose purpose is to prevent worry or discomfort rather than to give pleasure or satisfaction [8]. The individual performs the compulsions in order to reduce the distress caused by the obsessions and/or to prevent the dreaded event from occurring, although they are not realistically linked to the event and are clearly excessive. Though the degree of insight may differ, OCD sufferers frequently recognize them as useless and unhelpful in averting a feared consequence [6]. The average age of onset of the disorder is 19.5 years [9], although two incidence peaks with a bimodal distribution are recognized: the first occurring between 7 and 12 years, more typical of males, and the second around the age of 21, more typical of females [5,10]. Onset after the age of 40 is considered unusual [8,11]. The course of the disease is generally either chronic, involving the persistence of symptoms over time, the severity of which may fluctuate, with possible phases of exacerbations and incomplete remissions, or episodic, with symptoms appearing only during the episode with spontaneous or pharmacological regression [12]. Generally, if untreated, OCD has a chronic course with a rate of spontaneous remission between 5% and 10% [8,13,14], while a progressive worsening of symptoms was observed in only 8–10% of patients [15,16,17]. Individuals with OCD often present other comorbid psychiatric disorders with important clinical and therapeutic consequences [5]. The disorders most frequently found in comorbidity in these subjects are anxiety disorders [6] and, in particular, social anxiety, followed by specific phobias, panic disorder, post-traumatic stress disorder (PTSD) and generalized anxiety disorder [5,18,19].

ASD is a common neurodevelopmental disorder, with a prevalence ranging from 0.9% [20] to 1.85% [21], and whose core features include deficiencies in social communication and social reciprocity, repetitive behavior and narrow interests [6]. ASD is recognized as a widely heterogenous disorder, with various subtypes, different onsets and diverse developmental phases and manifestations [22]. Individuals diagnosed with ASD may exhibit distinct cognitive styles compared to those without the disorder. These differences may include a tendency towards systematizing, or the need to analyze systems to comprehend and forecast results, as well as heightened attention to detail and diminished focus on general information [23]. Alongside the main manifestations of ASD, comorbid psychiatric disorders are quite common, increasing disability and making diagnosis and treatment more difficult [24,25,26]. Subjects with ASD range widely in their functional abilities, from those who can live independently to those who may not be able to function well in nearly every aspect of life, so that a more recent conceptualization of ASD views it as a heterogeneous condition whose manifestations are distributed over a continuum that ranges from the least severe to the most extreme manifestations. Recent studies on ASD have highlighted the need for looking at both the full-blown clinical forms and milder, subclinical forms of the disorder, which seem to be spread over a continuum from the general to the clinical population [27,28,29,30,31]. First-degree relatives of individuals with ASD were the first to be examined for subthreshold autistic features, also known as the “broad autism phenotype” [32,33]. Nevertheless, further investigation has revealed other populations, such as psychiatric patients with various disorders and probands of autistic subjects [33]. In particular, to this date, many studies have highlighted a higher prevalence of autistic traits among subjects with bipolar disorder (BD), where they have also been reported to be correlated with greater depressive symptoms and higher prevalence of suicidal thoughts or behaviors across the lifetime [34]. Similarly, subjects with borderline personality disorder (BPD) reported higher autistic traits than the general population, which also, in this case, are associated with suicidality and lifetime exposure to physical and/or sexual abuse [35,36]. Moreover, particularly timely is the hypothesis of a correlation between feeding and eating disorders (FED), in particular AN, and ASD. Indeed, autistic traits have been frequently reported among FED patients and at the same time atypical eating behaviors are common in ASD subjects, leading numerous authors to the suggestion of conceptualizing FEDs as a possible psychopathological trajectory of a neurodevelopmental alteration wherein the female gender is one of several risk factors [37,38]. In this framework, recently growing interest has been dedicated to the concept of a relationship between orthorexia nervosa (ON) and autistic traits, due to the great similarities that the first shares with AN, showing a significant overlap between ON and autism spectrum psychopathology [39,40]. Lastly, numerous authors have highlighted the overlap between ASD and catatonia, both from a clinical and pathophysiological perspective [41]. Interestingly, in addition to subjects with other psychiatric disorders, high autistic traits were also detected in students of scientific courses [42,43]. In this context, subthreshold autistic traits are of particular relevance because they have a detrimental effect on quality of life and are a significant risk factor for the emergence of suicidal thoughts and behaviors, as well as a variety of psychiatric, and eventually somatic, disorders [43,44,45,46,47,48,49]. In spite of this, subthreshold autistic traits, or even milder clinical forms of ASD, still easily remain under-recognized and undertreated during adulthood, silently worsening the trajectory of other comorbid disorders [50].

In this framework, the Adult Autism Subthreshold Spectrum Questionnaire (AdAS Spetrum) was recently validated in order to assess the wide range on ASD manifestations, from the full-blown picture to the more subtle and subclinical traits, also including the atypical presentations, and other aspects associated with the primary symptoms [27,29].

At the same time, due to the presence of repetitive behaviors in both ASD and OCD, some authors have proposed a potential correlation between the two disorders [51,52,53,54,55,56]. This link has long been recognized and debated, with some authors arguing that these disorders frequently occur comorbid but distinct [57] while others instead believe that they are part of the same spectrum [58,59]. A large study conducted on the Danish population found that subjects with OCD have a four times greater risk of a subsequent diagnosis of ASD, just as subjects who received a first diagnosis of ASD had a twice greater risk of receiving a subsequent OCD diagnosis [60]. The prevalence of OCD is estimated to range from 4.9% to 37.2% in children and adolescents diagnosed with ASD, and from 7% to 24% in adults with ASD [24,61,62]. On the other hand, subjects with OCD not only share a higher risk of reaching the diagnostic threshold for ASD [56,60,63], but also manifest significantly higher autistic traits which make the manifestation of the disorder more severe [56,64,65]. However, most of these studies were conducted with children or adolescents, while the literature about adults remains scant [59,66,67,68]. In addition, despite the studies conducted, it is believed that the true prevalence rates of ASD or autistic traits in OCD remain underestimated due to the similarities of symptoms between the two disorders [59]; in fact, the restricted interests and repetitive behaviors of ASD may appear similar to the obsessions and compulsions of OCD, and, in both cases, these symptoms lead to social and functional impairments [69]. For these reasons, the differential diagnosis can be complex, although identifying the key characteristics that distinguish the two disorders remains essential to allow effective diagnoses and provide the right treatment opportunities [5].

To this date, studies evaluating autistic traits in OCD samples are still limited and mainly focused on children and adolescents. However, even in adults these traits can play a fundamental role in defining the psychopathological trajectory and response to treatment. Indeed, in OCD subjects, a poor response to treatment is highly correlated with psychiatric comorbidities, including ASD and autistic traits. However, because these characteristics were thought to be personality disorders or indications of OCD, they were typically ignored in treatment plans. As a result, ASD characteristics were disregarded, which might have led to OCD treatment resistance. In this framework, we aimed to explore not only the prevalence and correlates of autistic traits in a sample of adult subjects diagnosed with OCD and healthy controls (HC), but also to explore which possible autism dimensions are statistically predictive of higher OCD symptoms, in order to highlight dimensions that can lead to the hypothesis of the presence of autistic traits worthy of investigation.

## 2. Materials and Methods

Data were collected between September 2022 and March 2023 at the Psychiatric clinic of the University of Pisa.

### 2.1. Study Sample and Procedure

The sample assessed was composed of 110 subjects belonging to two diagnostic groups: 55 subjects belonged to the OCD group and 55 belonged to the HC group. The OCD group was recruited from of in- and out-patient at the Psychiatric Clinic of the University of Pisa, while the HC subjects were recruited from healthcare and paramedical personnel following a sex- and gender-matched criteria. All subjects’ ages ranged from 18 to 70 years old. The diagnoses of OCD and the absence of mental disorders among HC were confirmed using the Structured Clinical Interview for DSM-5, Research Version (SCID-5-RV) [70]. Subjects that met the following criteria were excluded: age under 18 years old, presence of a language or intellectual impairment that could compromise the examinations, mental disabilities, lack of collaboration skills or persistent psychotic symptoms.

This study was conducted in accordance with the Declaration of Helsinki. All recruitment and assessment procedures were approved by the Ethics Committee of the Azienda Ospedaliero-Universitaria of Pisa, on the 19 January 2023, with the approval code “23326_Dell’osso”. The procedures were comprehensively explained to the recruited subjects who then gave their written informed consent.

The participants did not receive any compensation for taking part in this study.

### 2.2. Measures

Assessment procedures included the SCID-5-RV [70], the Obsessive–Compulsive Spectrum—Short Version questionnaire (OBS-SV) and the Adult Autism Subthreshold Spectrum (AdAS Spectrum).

#### 2.2.1. The Structured Clinical Interview for DSM-5 Disorders (SCID-5)

The SCID-5 is the gold standard structured clinical interview for determining whether severe mental illnesses are present according to the DSM-5 [70]. It consists of ten separate modules, each with a different set of questions that lead the interviewer through evaluating whether symptoms meet the diagnostic criteria. The questions are arranged in accordance with the corresponding diagnostic manual (DSM-5).

#### 2.2.2. Obsessive–Compulsive Spectrum—Short version (OBS-SV)

The OBS-SV is a self-report questionnaire employed to assess both the typical symptoms and prototypic symptoms of OCD as well as unusual manifestations, temperamental traits and other clinical and subclinical aspects. The questionnaire consists of 139 items with dichotomous answers, organized in 6 domains and 2 appendices; the score is calculated by counting the number of positive responses. The domains include Doubt, Hypercontrol, Temporal dimension, Perfectionism, Repetition and automation and Obsessive themes, while the appendices cover the Childhood and adolescence and Impulsivity and loss of control. During the validation study, the OBS-SV exhibited excellent internal consistency, and test–retest reliability (Kunder-Richiardson coefficient = 0.964, ICC = 0.998), as well as a significant convergent validity with other OCD measures [71].

#### 2.2.3. Adult Autism Subthreshold Spectrum (AdAS Spectrum)

The AdAS Spectrum is a self-report tool made of 160 items, used to evaluate patients without intellectual or linguistic problems for a wide range of autism spectrum symptoms. The questionnaire is divided into seven domains: Childhood and adolescence, Verbal and nonverbal communication, Empathy, Inflexibility and adherence to routine, Restricted interests and rumination and Hyper- and hyporeactivity to sensory input. The responses to the different questions are binary (yes/no), and the scores for the appendices and single domains are calculated by totaling the number of yes responses. The validation study revealed a high internal consistency, great test–retest reliability (Kunder-Richiardson coefficient = 0.964, ICC = 0.976), and convergent validity with other dimensional measures of autism spectrum [27,29].

### 2.3. Statistical Analysis

Chi-square and the Student’s *t*-test were used to compare sociodemographic factors between groups. The results of the two diagnostic groups on the OBS-SV and AdAS Spectrum questionnaires were compared using the Student’s *t*-test. The pattern of correlations between the scores recorded on the two psychometric instruments within the OCD and HC participants was assessed using Pearson’s correlation coefficient. Then, using OBS-SV scores as the dependent variable and AdAS Spectrum total and domain scores as independent variables, linear regression analyses were carried out to determine which AdAS Spectrum domains were statistically predictive of OBS-SV scores in the sample. A K-Means cluster analysis based on the nine AdAS Spectrum domains was performed in order to evaluate the specific distribution of autistic traits in the sample. Then, a discriminant analysis was performed to confirm the validity of the group differentiation according to the cluster analysis.

Every statistical evaluation was performed with SPSS version 26.0 [72].

A flow diagram of the procedures and results is reported below:


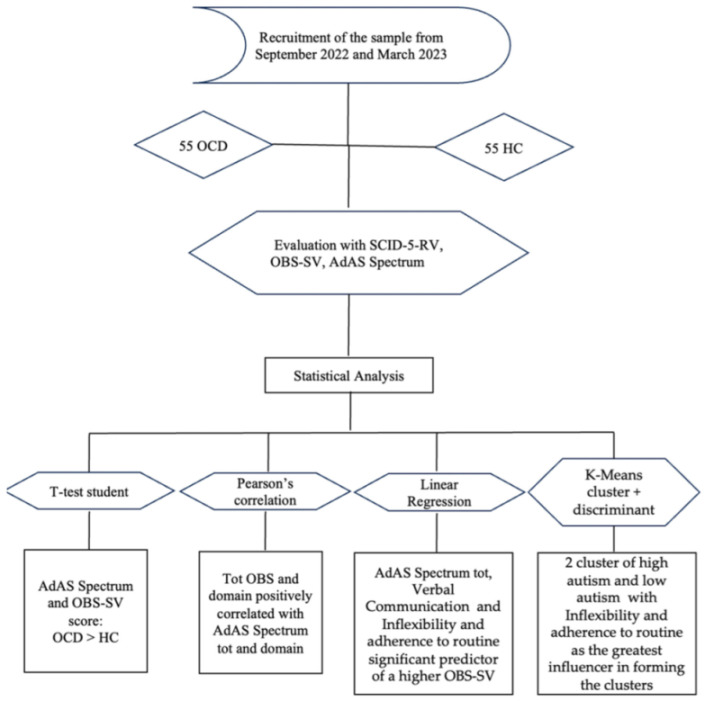


## 3. Results

The SCID-5 was used to confirm the diagnostic categorization of the OCD group and to verify the absence of mental disorders in the HCs. Subjects belonging to the HC group reported negative results in all modules while subjects belonging to the OCD group reported positive results in the OCD module. In the latter group, the interview was also used to exclude the presence of disorders belonging to the schizophrenia spectrum.

The OCD sample reported a mean age of 39.98 years (±11.27) and consisted of 25 (45.5%) males and 30 (54.5%) females. The HC group showed a mean age of 37.95 years (±12.65) and consisted of 27 (49.1%) males and 28 (50.9%) females, as reported in Table 1. Regarding the educational level, 13 (11.8%) subjects reported to having a master’s degree, 42 (38.2%) subjects reported having a degree, 43 (39.1%) subjects reported having graduated, 10 (9.1%) subjects had a middle school certificate, 1 (0.9%) subject did not finish the elementary school and 1 (0.9%) subject preferred not to disclose. Regarding the occupational role, 17 (15.5%) subjects were students, 24 (21.8%) subjects were unoccupied, 22 (20%) subjects were housewives, 31 (28.2%) subjects were employed, 3 (2.7%) subjects were retired and 13 (11.8%) subjects preferred not to disclose. Regarding marital status, 16 (14.5%) subjects reported living with their parents, 19 (17.3%) subjects were married, 23 (20.9%) subjects were unmarried, 9 (8.2%) subjects were divorced and 43 (39.1%) subjects preferred not to disclose.

Student *t*-test results showed that the OCD group scored significantly higher in all AdAS Spectrum and OBS-SV domains as well as in their total compared to the HC group, as reported in Table 2 and Table 3.

Results from the correlation analysis showed that, in the total sample, the total OBS-SV scores, as well as all OBS-SV domains scores, were significantly and positively correlated with all AdAS Spectrum domain and AdAS total scores. All correlation coefficients were medium to strong as reported in Table 4.

According to the regression analysis, the AdAS Spectrum total score was a significant predictor of a higher OBS-SV score (beta = 0.88; t = 17.15; *p* < 0.001) as shown in Table 5.

Results from a further linear regression analysis, with AdAS Spectrum domains as independent variables, showed that the AdAS Spectrum Verbal Communication (beta = 0.29; t = 2.76; *p* = 0.007) and Inflexibility and adherence to routine (beta = 0.37; t = 3.75; *p* < 0.001) domain scores were significant positive predictors of high OBS-SV total scores as reported in Table 6.

**Figure 1:**
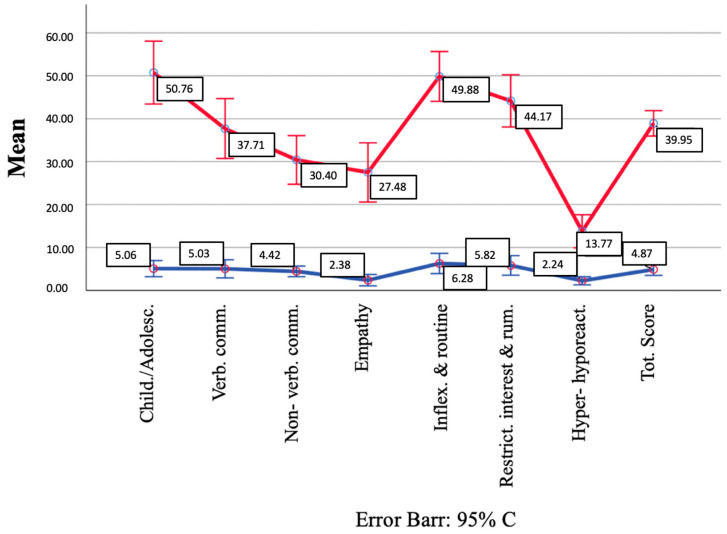
AdAS Spectrum domain scores in the two clusters identified.

Then, we defined the two clusters of subjects with a K-means cluster analysis: the high autism cluster (n = 47, 42.7%) and the low autism cluster (n = 63, 57.3%). The lines charts of Figure 1 show the mean values of the AdAS domain and total scores in the two clusters. The distance between the high autism and the low autism final cluster centers was 89.251. The average distance between cases and their classification cluster center was 29.44 ± 25.32. In the dispersion analysis, the Inflexibility and adherence to routine domain presented the greatest influence in forming the clusters (F = 236.61), while the Hyper– and hyporeactivity to sensory input domain had the lowest influence (F = 44.05) as shown in Table 7.

A discriminant analysis performed on the two clusters confirmed the results of the cluster analysis, as reported in Table 8.

Furthermore, the analysis confirmed the results obtained from the cluster analysis in relation to the identification of the weight of each AdAS Spectrum domain in discriminating the clusters as shown in the structure matrix reported in Table 9.

**Figure 2:**
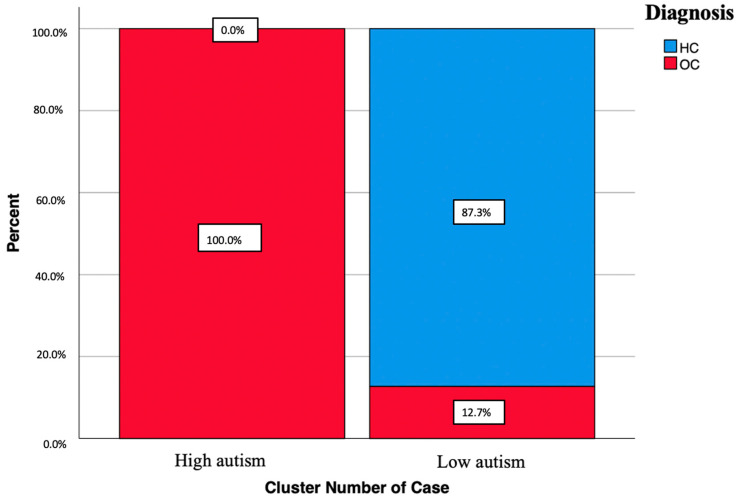
Diagnostic group composition on the basis of clusters.

Figure 2 shows the composition of clusters with respect to diagnostic groups.

## 4. Discussion

Our findings show that, in addition to scoring significantly higher than the HC group in the OBS-SV questionnaire, OCD subjects also reported a higher prevalence of positive answers in all AdAS Spectrum domains and in the questionnaire’s overall score. The results also highlighted a positive and strong correlation between all OBS-SV domains and AdAS Spectrum domains as well as between their total scores. These findings are coherent with previous studies and confirm the noteworthy prevalence of autistic features in OCD-affected adults. Indeed, previous studies have demonstrated a high prevalence of autistic traits in individuals with OCD and have shown that higher scores on ASD traits were also linked to the presence of other psychiatric comorbidities in OCD subjects [51,52,54,55,73]. Moreover, due to the presence of overlapping features between the two disorders, some authors argued that these disorders may be part of the same spectrum [58,59], ultimately supporting the hypothesis of a possible common neurodevelopmental basis for various psychiatric conditions. For instance, recent research suggested that they may share similar underlying origins, which could explain why they manifest together more frequently in the same person than what would be expected [74,75]. In particular, while some authors investigated possible shared genetic causes [75], others suggested common differences in white matter [74] in the dorsomedial pre-frontal cortex and in networks of neurons regulating the basal ganglia [76]. Additionally, studies have also demonstrated not only that characteristics of the two illnesses can co-occur and overlap, but also that higher autistic traits in this population are associated with the development of other comorbid disorders like tic disorders and attention deficit hyperactivity disorder [55].

In particular, the strongest correlation that emerged from our results regarded the OBS-SV Perfectionism domain and the AdAS Spectrum Inflexibility and adherence to routine domain. Interestingly, this result appears to be in line with presence of a strong cognitive inflexibility in both disorders that shapes the drive toward perfectionism in OCD and the tendency to repetitive behaviors in ASD [77,78,79,80]. In general, cognitive flexibility is defined as the capacity to adapt behavior to the requirements of a changing environment and, in more detail, to focus set shifting and task shifting tests [81,82]. Moreover, this cognitive structure and the resulting maladaptive or negative perfectionism represent a risk factor for the onset of suicidal ideation, depressive symptoms and anxiety [83,84]. Clinically, OCD sufferers find it challenging to switch between different thought processes in order to produce adaptive behavioral responses, particularly while their symptoms are present, indicating a pervasive deficit in cognitive flexibility [79,80,85,86,87]. Similarly, people with ASD show greater cognitive inflexibility compared to the general population, and those difficulties with executive functioning appear to be correlated with greater symptoms of both emotional and behavioral difficulties [88,89,90,91].

According to our results, the AdAS Spectrum total score and AdAS Spectrum Verbal Communication and Inflexibility and adherence to routine domains were significant positive predictors of higher scores in the OBS-SV. Interestingly, while being one of the hallmark symptoms of ASD, impairment in communication and social interaction has recently been addressed and described in OCD populations as well [92]. In particular, the deficiencies revolve around pragmatic competence which includes the application of both language and extralinguistic communication [93] and manifests through strange verbal interactions that affect the “clarity and relevance of the message being communicated,” inadequate disinhibition and poor expression [94]. While these mild pragmatic impairments have been suggested as characteristics of a wider autism phenotype given that they are more common in relatives of probands with autism [95,96], they can also pave the way for new research on the symptomatologic overlap between OCD and ASD. While evidence on shared alterations in social communication are still in their infancy, the finding of Inflexibility and adherence to routine domain scores being positive predictors of OCD symptomatology is in line with a substantial body of studies that found cognitive inflexibility as the primary shared background for the association between OCD and autistic features. As stated, cognitive inflexibility is the tendency to fixate on one’s own thoughts, beliefs or behaviors, and involves executive functioning [97]. This tendency limits adaptable problem-solving and the ability to shift focus from the present thought, making it difficult to transition from one thought or behavior to another [82,98]. On the neuropsychological level, cognitive flexibility is effectively disengaging from an activity or type of action and moving to another, while hindering the initial task response or perseverative response patterns. Regarding autism, cognitive rigidity is frequently linked to the restricted and repetitive behavior domain [99]. In particular, research on individuals with ASD has demonstrated a strong correlation between heightened severity of restricted and repetitive behavior and neuropsychologically evaluated aspects of executive functioning linked to cognitive inflexibility [77,78]. In this framework, repetitive behaviors, one of the main symptomatologic overlaps between OCD and ASD, may represent the behavioral expression of an inflexible cognitive tendency typical of both disorders.

Indeed, both in OCD and ASD repetitive behaviors refer to a series of actions that are performed repetitively and are viewed as strange or improper [100]. In OCD, obsessions are persistent, bothersome thoughts, frequently connected to themes of sexuality, religion or contamination, while compulsions are actions carried out as a way to compensate for these bothersome thoughts to reduce anxiety, and they include repetitive everyday chores such as checking or handwashing. Repetitive behaviors in ASD can range in kind and intensity, can be more complex, like insisting on sticking to a daily schedule, lining up objects or repeatedly watching the same video, or they can be stereotyped motor behaviors, like hand flapping, rocking and shaking fingers in front of their eyes [101]. Remarkably, while for both disorders these behaviors are time consuming and affect negatively global functioning, in OCD those recurrent habits are undesired and irritating and perceived as ego-dystonic, while repetitive behaviors in ASD are not always distressing; instead, they might be desired or consoling behaviors for those on the spectrum and ultimately perceived as ego-syntonic [102]. However, to date, research aiming to verify the differences between repetitive behaviors in ASD and OCD have yielded inconsistent findings [103,104], and due to this symptomatologic overlap, it can be difficult to distinguish between behaviors that indicate the presence of autistic traits in OCD subjects, resulting in frequent over- or underdiagnosis [105].

Furthermore, according to our analysis, two groups of significantly different severity with respect to autistic traits were identified, thanks to the application of a cluster analysis applied to the scores obtained in the AdAS Spectrum questionnaire, whose validity was further confirmed with a discriminant analysis. As expected, the high autism cluster was found to be more represented in the OCD group, whereas the low autism cluster was significantly more represented in the HC group, confirming the higher prevalence of autistic features in OCD subjects. The AdAS Spectrum domain that demonstrated the greatest influence in the differentiation of the clusters, according to both the discriminant analysis and the cluster analysis, was the Inflexibility and adherence to routine domain, once again confirming the role of this dimension as the main shared background for ASD and OCD. In conclusion, both ASD and OCD are crippling disorders that affect young people, whose co-occurrence can be extremely difficult to manage. According to a number of studies, people with co-occurring ASD or elevated autistic traits and OCD may have clinically different psychopathology and impairment from those with only one disorder. In light of this evidence, the early identification and evaluation of an underlying diagnosis of ASD or high autistic traits appears crucial to guarantee timely and accurate treatment and to improve the outcome, global prognosis and quality of life of the subject.

This association, again confirmed between OCD and autistic traits, may be due not only to overlapping diagnostic criteria but also to a shared etiology. For instance, genetic studies have linked chromosomes 7q, 1, 6, 19 and 15q11–13 to obsessive symptoms in autistic subjects [106,107,108] and have identified the serotonin transporter, tryptophan hydroxylase, glutamine transporter genes and a glutamate receptor gene as candidate genes in both OCD and ASD [109]. In addition, neuroimaging research has highlighted that gray matter volume of the left dorsolateral prefrontal volume is correlated with the severity of repetitive behaviors in autistic adults and could mediate repetitive behaviors related to OCD symptoms [110]. Similar research has showed a correlation between measures of autistic traits and regional gray matter volumes in the left dorsolateral prefrontal cortex and amygdala of OCD patients [111]. Even if, to this date, studies investigating the neuroanatomical overlap between OCD and ASD are still scarce, major research has demonstrated lower function and a smaller structure of the rostrodorsomedial prefrontal cortex in both ASD and OCD [112].

Ultimately, knowledge of the relationship between autistic features and OCD can also benefit the treatment programs proposed for patients. The National Institute for Health and Care Excellence (NICE) treatment guidelines suggest that adults with OCD should be offered cognitive behavior therapy (CBT), selective serotonin reuptake inhibitor (SSRI) therapy or combined SSRI/CBT treatment. However, OCD seems to have a high likelihood of recurrence and a low rate of remission, possibly due to the presence of psychiatric comorbidities, including ASD [113,114].

A number of studies assessing cognitive behavioral therapy (CBT) in the treatment of OCD with co-occurring ASD have found that participants generally benefited from treatment [115]. Conversely, while some research that compared OCD patients without ASD to individuals with co-occurring ASD saw a much smaller reduction in OCD symptoms during therapy and a worse rate of remission after treatment [116], other research has lacked to report such a difference [117]. Globally, initial results indicate that adolescents with co-occurring ASD can effectively treat their OCD with intense CBT [118]; however, to learn more about how subclinical autism features affect the effectiveness of CBT in OCD-affected children and adolescents, more research is necessary.

Important limitations should be taken into account while evaluating this study. Initially, the cross-sectional design precluded us from drawing conclusions regarding the temporal or causal link among the variables under investigation. Furthermore, the limited sample size restricts the generalizability of our findings. Although the age of onset for OCD is typically in adolescence and early adulthood, ASD shows a wider range in the age of onset; as a result, it was decided to include in the study sample subjects with an age ranging between 18 and 70 years old in order to include a vast range of manifestations of both disorders. However, in light of the different age-related manifestations and prevalence rates of these two disorders, which underline the complex nature of their interactions throughout life, the results of this study must be interpreted in consideration of the potential biases introduced by the related variations in age with respect to the symptoms and progression of the disease. Furthermore, although K-Means cluster analysis represents a prevalent method for clustering, it has several inherent limitations such as sensitivity to the initial selection of cluster centroids, susceptibility to outliers and the need for a homogeneous distribution, which could introduce biases into the analytical results. Also, although the AdAS Spectrum and the OBS-SV assess a wide range of manifestation ranging from the classic ones to the more atypical and subtle, the self-report nature of those questionnaires may ultimately lead to an over or underestimation of the symptomatology. Globally, more research is required, perhaps using a longitudinal approach, to elucidate the part played by underlying autistic features in the emergence and maintenance of OCD symptoms.

## 5. Conclusions

Our findings support the association between OCD and autism traits in the adult population. They also describe the high prevalence of autism traits in adults with OCD, as well as the strong correlation between many essential characteristics of the two conditions and the predictive function of autism traits in relation to more severe OCD symptoms. From a broader viewpoint, our findings may support the rising hypothesis of a possible shared neurodevelopmental basis for some psychiatric conditions, according to which different psychiatric illnesses, such as OCD in this case, may result from a neurodevelopmental alteration similar to the one associated with ASD, and might potentially be seen as a particular phenotype on the autistic spectrum [28,50,119].

## Figures and Tables

**Table 1:** Age and sex in the overall sample and comparison between diagnostic groups.

		OCD	HC		
		Mean ± SD	Mean ± SD	t	*p*
**Age**		39.98 ± 11.27	37.95 ± 12.65	−0.892	<0.375
		**n (%)**	**n (%)**	**Chi-square**	***p***
**Sex**	**M**	25 (45.5%) ^a^	27 (49.1%) ^a^	0.146	<0.702
	**F**	30 (54.5%) ^a^	28 (50.9%) ^a^		

Each superscript letter denotes a subset of categories whose column proportions do not differ significantly from each other at the 0.05 level.

**Table 2:** Comparison of AdAS Spectrum scores among the diagnostic groups.

AdAS Spectrum Scores	OCD Group Mean ± SD	HC Group Mean ± SD	t	*p*
** Childhood/Adolescence **	9.636 ± 5.481	0.690 ± 1.120	−11.857	<0.001
**Verbal communication**	6.345 ± 4.204	0.490 ± 0.604	−10.222	<0.001
**Non-verbal communication**	7.709 ± 5.408	0.981 ± 1.178	−9.014	<0.001
** Empathy **	2.963 ± 2.755	0.181 ± 0.474	−7.379	<0.001
**Inflexibility and adherence to routine**	19.873 ± 9.026	1.545 ± 1.686	−14.817	<0.001
**Restricted interest and rumination**	8.472 ± 4.630	0.854 ± 1.253	−11.778	<0.001
**Hyper- and hyporeactivity to sensory input**	2.145 ± 2.138	0.290 ± 0.533	−6.242	<0.001
**Total Score**	57.145 ± 19.709	5.036 ± 4.185	−19.180	<0.001

**Table 3:** Comparison of OBS-SV scores among the diagnostic groups.

OBS-SV Scores	OCD Group Mean ± SD	HC Group Mean ± SD	t	*p*
** Doubt **	5.163 ± 1.960	0.890 ± 1.242	−13.653	0.001
** Hypercontrol **	18.236 ± 5.360	2.690 ± 3.436	−18.105	<0.001
** Temporal dimension **	3.581 ± 1.750	0.600 ± 1.148	−10.565	0.002
** Perfectionism **	8.054 ± 3.412	1.200 ± 1.310	−13.906	<0.001
** Repetition and automation **	3.454 ± 2.035	0.472 ± 0.959	−9.829	<0.001
** Obsessive themes **	9.472 ± 4.354	1.018 ± 1.239	−13.850	<0.001
**Total Score**	47.963 ± 10.148	6.872 ± 7.295	−24.381	0.001

**Table 4:** Pearson’s correlations coefficients (r) among OBS-SV domain scores and AdAS Spectrum scores in the total sample.

	Child./Adolesc.	Verb. Comm.	Non- Verb. Comm.	Empathy	Inflex. and Routine	Restrict. Interest and Rum.	Hyper-Hyporeact.	Tot. Score
** Doubt **	0.645 *	0.761 *	0.631 *	0.494 *	0.566 *	0.562 *	0.249 *	0.711 *
** Hypercontrol **	0.743 *	0.581 *	0.487 *	0.553 *	0.764 *	0.714 *	0.515 *	0.802 *
** Temporal dimension **	0.710 *	0.771 *	0.654 *	0.558 *	0.626 *	0.561 *	0.267 *	0.758 *
** Perfectionism **	0.643 *	0.439 *	0.384 *	0.604 *	0.853 *	0.697 *	0.494 *	0.778 *
** Repetition and automation **	0.518 *	0.565 *	0.480 *	0.585 *	0.582 *	0.556 *	0.335 *	0.650 *
** Obsessive themes **	0.593 *	0.637 *	0.653 *	0.484 *	0.586 *	0.550 *	0.303 *	0.696 *
**Total Score**	0.756 *	0.687 *	0.607 *	0.621 *	0.792 *	0.724 *	0.460 *	0.855 *

* Significant correlation for *p* < 0.05.

**Table 5:** Linear regression analysis with OBS-SV total score as a dependent variable and AdAS Spectrum total score as the independent variable in the total sample.

	b (SE)	BETA	t	*p*	CI 95%	
					Lower Bound	Upper Bound
*constant*	7.379 (1.615)	0.885	4.570	<0.001	4.179	10.579
**AdAS Spectrum**	0.645 (0.038)		17.146	<0.001	0.570	0.719
**Total Score**						

R square = 0.731; Adjusted R square = 0.729.

**Table 6:** Linear regression analysis with OBS-SV total score as a dependent variable and AdAS Spectrum domains as independent variables in the total sample.

	b (SE)	BETA	t	*p*	CI 95%	
					Lower Bound	Upper Bound
*constant*	7.397 (1.636)	-	4.521	<0.001	4.152	10.643
**Verb. comm.**	1.539 (0.556)	0.288	2.759	0.007	0.432	2.645
**Inflex. and routine**	0.741 (0.196)	0.371	3.784	<0.001	0.352	1.129

R square = 0.747; Adjusted R square = 0.729.

**Table 7:** K-means cluster analysis features. Dispersion analysis.

AdAS Spectrum Z Scores	Cluster Mean Square (SE)	F	*p*
** Child./Adolesc. **	56,207.604 (296.527)	189.553	<0.001
** Verb. comm. **	28,748.850 (281.579)	102.099	<0.001
** Non- verb. comm. **	18,159.432 (174.222)	104.232	<0.001
** Empathy **	16,960.504 (251.213)	67.514	<0.001
**Inflex.** **and routine**	51,172.548 (216.277)	236.606	<0.001
**Restrict. interest** **and rum.**	39,597.764 (229.338)	172.661	<0.001
**Hyper-hyporeact.**	3576.239 (81.190)	44.048	<0.001

**Table 8:** Discriminant analysis.

**Eigenvalues** **Function**	**Eigenvalue**	**% of Variance**	**Cumulative %**	**Canonical Correlation**
1	3.907	100.0	100.0	0.892
**Wilks’ Lambda Test of Function(s)**	**Wilks’ Lambda**	**Chi-square**	**df**	**Sig.**
1	0.204	166.218	7	<0.001

**Table 9:** Discriminant analysis: structure matrix.

AdAS Spectrum Z Scores	Function 1
**Inflexibility and adherence to routine**	0.721
**Childhood/Adolescence**	0.577
**Restricted interest and rumination**	0.573
**Verbal communication**	0.498
**Non-verbal communication**	0.439
**Empathy**	0.359
**Hyper– and hyporeactivity to sensory input**	0.304

## Data Availability

The raw data supporting the conclusions of this article will be made available by the authors, without undue reservation. The data are not publicly available due to their containing information that could compromise the privacy of research participants.
